# From Pledge to Practice: A Call for FIGO/WHO to Issue Harmonized, Consolidated Abortion Care Guidelines

**DOI:** 10.1002/puh2.70206

**Published:** 2026-03-02

**Authors:** Abraham Fessehaye Sium, Don Eliseo‐III Lucero‐Prisno

**Affiliations:** ^1^ Department of Obstetrics and Gynecology St. Paul's Hospital Millennium Medical College Addis Ababa Ethiopia; ^2^ Department of Global Health and Development, Faculty of Public Health and Policy, London School of Hygiene and Tropical Medicine London UK

**Keywords:** FIGO, quality abortion care, reproductive health, safe abortion, sexual and reproductive health (SRH), unsafe abortion, World Health Organization (WHO)

## Abstract

October 5, 2025, saw the launch of first‐ever consolidated postpartum hemorrhage (PPH) management guidelines issued by three global health agencies—World Health Organization (WHO), Federation International of Gynecologists and Obstetricians (FIGO), and International Confederation of Midwives (ICM)—at the FIGO congress in Cape Town, South Africa. This landmark XXV FIGO 2025 congress also hosted inauguration of two vital pledge walls—“End PPH” and “Safe abortion saves women. Denial takes them.” These pledge walls represent a commitment to initiatives of end two leading causes of maternal mortality at global level, which are PPH and unsafe abortion. We call on WHO and FIGO—in collaboration with Society of Family Planning (SFP) and other guideline‐setting bodies—to convene a similar harmonization and process and issue a consolidated, consistent abortion care guidelines to reduce contradictory recommendations and improve uptake of evidence‐based recommendations.

## 1 Introduction

Among the events that make the recent Federation International of Gynecologists and Obstetricians (FIGO) 2025 congress held in Cape Town historic is the inauguration of two vital pledge walls, on which several global agencies’ leaders, scientific society delegates, sexual and reproductive health (SRH) advocates, researchers, physicians, midwives, non‐governmental health agencies representatives, and policy makers signed their support to end two leading drivers of maternal mortality—postpartum hemorrhage (PPH) and unsafe abortion—globally. Interestingly, the first pledge “End PPH” was accompanied by the launch of a consolidated new guideline by global health agencies (FIGO, World Health Organization [WHO], and International Confederation of Midwives [ICM]) on first day of the congress, before the mentioned attendees put their signatures for it [1]. This unprecedented harmonization and partnership sourced from a deeper understanding that fragmented/contradictory guidance across these leading global health agencies reduces uptake of recommendations and creates uncertainty [2]. This strategic unification of addressing fragmentation and contradictory guidance of recommendations for PPH through FIGO/WHO/ICM consolidated PPH guidelines has been hailed as one of the most pragmatic tools to accomplish the “End PPH pledge.” The same FIGO 2025 pledge for ending unsafe abortion creates a momentum for a parallel abortion care guidelines consolidation. Therefore, WHO/FIGO (and other partners) should issue harmonized, consolidated abortion care guidelines.

## 2 What Does 2025 FIGO/WHO Partnership for Consolidated PPH Guidelines Demonstrate?

The direct take‐home message from the ceremonial of FIGO/WHO partnership at FIGO 2025 regarding abortion care guidelines is that the same tool (launching consolidated guidelines by the leading global health organizations—WHO and FIGO) can be used to attain the second pledge—“Safe abortion saves women. Denial takes them.” Essentially, what seems like unprecedented collaboration (launching the consolidated PPH guidelines) between WHO and FIGO has been exercised in different forms over the past three decades in support of the latter pledge. SRH for all is achievable provided that cost‐effective interventions are properly scaled up; political commitment is revitalized; and financial resources are mobilized, rationally allocated, and more effectively used [3]. Laying the founding work and subsequent advancement of SRH and rights as we know it today was carried out by FIGO and WHO with substantial health policy and clinical care reforms by several WHO member countries in response to the call for evidence‐based advocacy and SRH care reforms by these two world biggest health agencies. Matching the role of the current FIGO president (Professor Anne Kihara President) in launching the new PPH guidelines through creation of successful collaboration between FIGO and WHO, history remembers former FIGO president, Professor Mahmoud Fathalla, as the driving force behind the shift from a traditional, disease‐focused approach to reproductive health to one that emphasized women's overall well‐being, rights, and context [4, 5]. His leadership at the UN's Special Programme on Human Reproduction was instrumental in this conceptual shift, advocating for a more holistic definition of reproductive health [6], following which in 2004 WHO published a reproductive health strategy—ratified by 191 Member States at the Fifty‐Seventh World Health Assembly [7].

## 3 What Is Needed Now for Abortion Care Guidelines?

There is a concern over increasing restrictions to abortion care globally, both through more hostile abortion policy and withdrawal of funding, which would force more women to resort to unsafe abortion. On the other hand, there is a need to access  quality consolidated guidance to improve quality and safety of abortion care  across low–middle‐income countries (LMICs) [8]. Like the recommendations for PPH across the guidelines by the  international organizations (before their unison through the issuance of the consolidated guidelines on October 5), some of the current recommendations for abortion care across similar global organizations (FIGO, WHO, Society of Family Planning [SFP]/SFMM) are contradictory to one another. For example, the recommendations for abortion at later gestation (after 24 weeks) are different across these guidelines [8, 9, 10, 11, 12]. Addressing such and other differences could improve the uptake of safe abortion care recommendations across LMICs. Hence, formulating and publishing quality abortion care guidelines by replicating the unprecedented collaboration between WHO and FIGO and inviting other organizations (SFP and Society of Fetal‐Maternal Medicine), as demonstrated by the PPH guidelines, is vital and timely.

## 4 Conclusion

Unsafe abortion continues to drive avoidable maternal deaths globally, but the issue extends beyond safety alone: access to quality abortion care is central to reproductive autonomy, dignity, and human rights. A coordinated WHO–FIGO effort—modeled on the PPH consensus—could strengthen shared guidance and advocacy, improving both health outcomes and the realization of reproductive health rights.

## Author Contributions

Abraham Fessehaye Sium and Don Eliseo‐III Lucero‐Prisno  developed the concept of the article and wrote the manuscript. Both authors checked the manuscript against intellectual contents and approved the final version for publication.

## Funding

The authors have nothing to report.

## Ethics Statement

The authors have nothing to report.

## Conflicts of Interest

The authors declare no conflicts of interest.

## Data Availability

The authors have nothing to report.
